# Effect of SEC III protocol on upper airway dimensions in growing class III patients: a retrospective study

**DOI:** 10.1186/s12903-023-03613-8

**Published:** 2023-11-08

**Authors:** Ahmed R. Elkalza, Yomna M. Yacout

**Affiliations:** Department of Orthodontics, Faculty of Dentistry, Alexandria University, Champollion St, P. O. Box: 21521, Alexandria, Alexandria Egypt

**Keywords:** Cephalometrics, Chin cup, Class III malocclusion, Class III splint, Class III intraoral elastics, Oropharynx, Nasopharynx

## Abstract

**Background:**

The primary objective of the study was to evaluate the effects of SEC III (Splints, Class III Elastics, and Chin cup) protocol on the upper airway dimensions using lateral cephalometric radiographs. The secondary objectives were to evaluate the skeletal and dental effects of the SEC III protocol using lateral cephalometric radiographs.

**Methods:**

The pre- and post-treatment lateral cephalometric radiographs of 24 patients treated using the SEC III protocol were used to address the aim of the study. Children in the pre-pubertal (CS-1 or CS-2) or circumpubertal (CS-3 or CS-4) skeletal maturation stage and having class III dentoskeletal malocclusion were included in the study. Patients with a history of previous orthodontic treatment, maxillofacial surgery or trauma, tonsillectomy, adenoidectomy, or craniofacial malformations were excluded. The pre-treatment and post-treatment lateral cephalometric radiographs were traced, then airway measurements, skeletal measurements, and dental measurements were performed. The results were analysed using paired samples t-test or Wilcoxon signed rank test according to the data normality.

**Results:**

Data of 6 males and 18 females were analysed (Mean age = 11.21 ± 1.02 years). Duration of active treatment was 5.75 ± 1.03 months. Treatment using SEC III protocol resulted in a significant increase in ANB angle (2.92 ± 1.50 degrees, p < 0.001) and Wits appraisal (3.31 ± 1.99 mm) (p < 0.001). The increase in the mandibular plane angle (0.75 ± 1.42 degrees, p = 0.02) and the maxillary length (2.29 ± 2.69 mm, p < 0.001) was statistically significant. Contrarily, the mandibular length did not change significantly (p = 0.10). The maxillary incisors were significantly proclined (4.38 ± 4.28 degrees; p < 0.001), while the mandibular incisors were significantly retroclined (-5.79 ± 6.21 degrees; p < 0.001) following treatment. The change in the nasopharyngeal airway and the retropalatal airway was not statistically significant. The middle and inferior pharyngeal space (retroglossal airway) significantly decreased by 1.33 ± 1.97 mm (p = 0.003) and 1.96 ± 2.48 mm (p = 0.001), respectively.

**Conclusions:**

Early class III correction using SEC III protocol reduced the retroglossal airway dimensions but did not affect the nasopharyngeal and retropalatal airway dimensions. Correction of the class III dentoskeletal relationship was obtained through both skeletal and dental changes.

## Background

An Angle class III malocclusion on a skeletal class III base is one of the most challenging types of malocclusions. The skeletal class III relationship may arise from a retrognathic maxilla, a prognathic mandible, or a combination of both [1]. The discrepancy in jaw size may result in aesthetic [2], speech [3], and functional impairment [4]. Early diagnosis of the skeletal discrepancy allows timely management by the orthodontist. Treatment of skeletal class III malocclusion in growing patients improves both function and aesthetics, and consequently boosts the patients’ self-esteem and improves their psychological state [5]. In addition, early intervention to correct the skeletal discrepancy may reduce the need for more aggressive treatment approaches at an older age [6].

Interceptive treatment for skeletal class III may be performed using a wide variety of treatment approaches [7, 8]. One of these approaches is the SEC III protocol which involves the simultaneous use of upper and lower removable splints, class III elastics and a chin cup [9]. It has been previously shown to result in significant orthopaedic effects on the maxilla and the mandible [10, 11].

The size of the maxilla and the mandible has been shown to correlate with the pharyngeal airway dimensions [12]. Hence, the skeletal effects of SEC III may be accompanied by a positive or negative effect on the airway dimensions. Altering the dimensions of the airway may influence the patients’ breathing mode [13], hence their craniofacial development [14]. Moreover, changing the airway dimensions may help alleviate breathing disorders [15]. Although the dentoalveolar and skeletal effects of SEC III have been studied before [9, 10, 16, 17], current evidence regarding its effect on the airway dimensions is lacking.

Airway dimensions can be assessed using different imaging techniques, one of which is lateral cephalometric radiography [18]. Lateral cephalograms are obtained routinely as a part of orthodontic diagnostic procedures. Therefore, cephalometric analysis allows measurement of the airway dimensions without exposing the patient to additional radiation.

Hence, the aim of the current study was to evaluate the effects of the SEC III protocol on the upper airway dimensions, the skeletal dimensions, and the dental parameters using lateral cephalometric radiographs. The null hypothesis of the current study was that the use of SEC III protocol to treat a developing skeletal class III malocclusion does not affect the airway dimensions, the skeletal dimensions, or the dental parameters.

## Methods

The objectives of the study were achieved using a retrospective study design. Ethical approval was acquired from the Institutional Review Board of the Faculty of Dentistry, Alexandria University (IORG:0008839, approval no. 0581–01/2023) before commencing the study. Pre- and post-treatment orthodontic records were selected from the archives of the Orthodontic Department at the Faculty of Dentistry, Alexandria University, and from the archives of the private practice of one of the authors (A.E.) according to the following inclusion criteria: 1- Class III dentoskeletal malocclusion (ANB angle ≤ 0°, class III molar relationship, anterior crossbite or edge to edge incisor relationship), 2- Cervical stage (CS) showing pre-pubertal (CS-1 or CS-2) or circumpubertal (CS-3 or CS-4) skeletal maturation according to the cervical vertebral maturation (CVM) method [19], 3- Treatment using the SEC III protocol [9] without any modifications [20], 4- Availability of pre-treatment (T1) and post-treatment (T2) lateral cephalometric radiographs of good quality. Patients treated using the modified SEC III protocol with an expansion screw [20], and patients with a history of previous orthodontic treatment, maxillofacial surgery, facial trauma, tonsillectomy, adenoidectomy, or craniofacial malformations were excluded. The patients’ parents or guardians provided informed consents for the use of their data for research purposes.

**Fig. 1:**
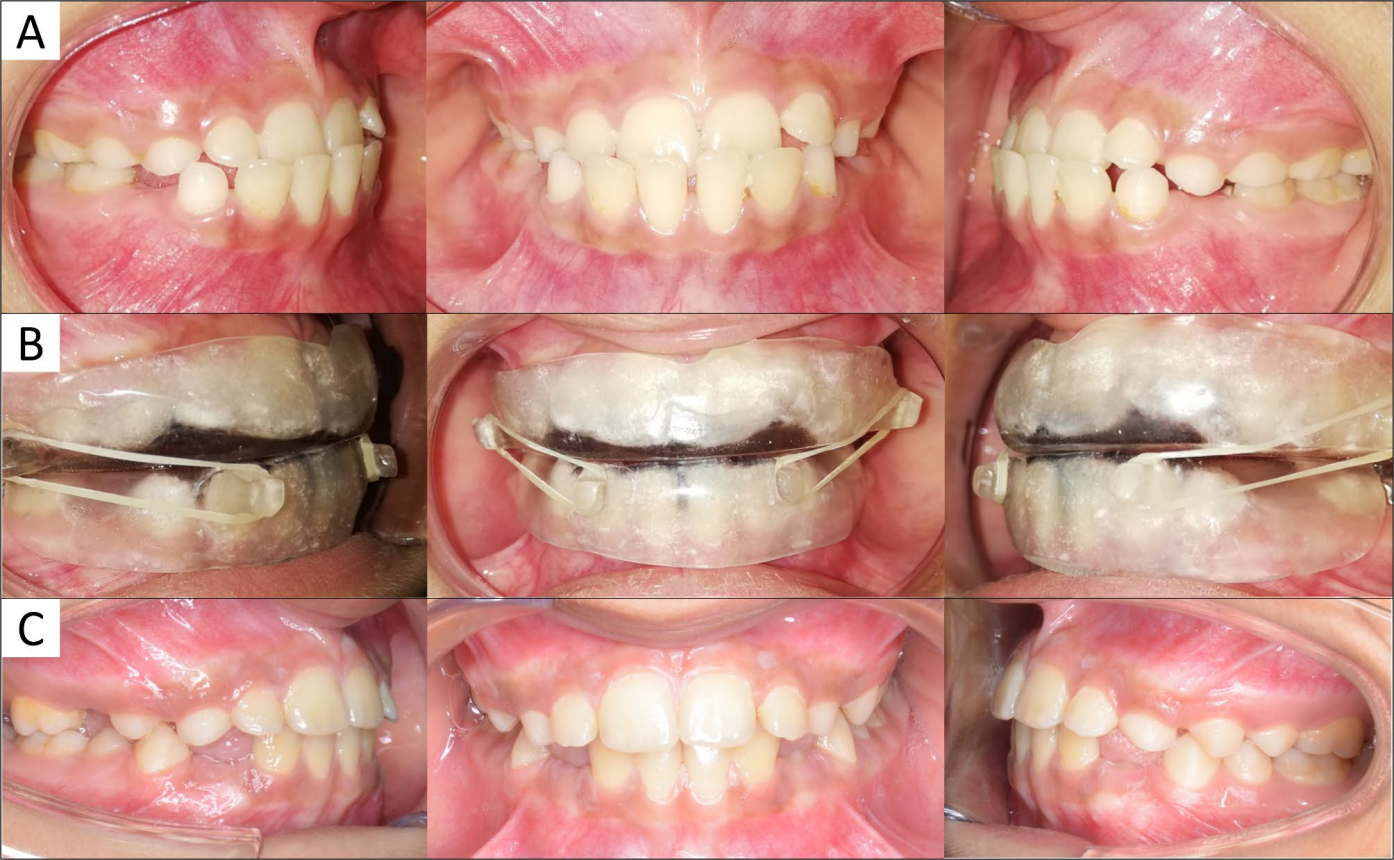
Intraoral photographs of a patient treated using SEC III protocol: **(A)** Pretreatment; **(B)** Class III splint and class III elastics; **(C)** Posttreatment

Treatment using SEC III protocol involved placement of class III elastics on upper and lower removable occlusal splints in addition to a chin cup attached to an occipital headgear. The buccal, lingual, and occlusal surfaces of both the upper and lower dentition were fully covered by the acrylic splints. The occlusal surfaces of the splints were made flat. Hooks for the class III elastics were placed bilaterally in the upper splint distal to the upper first molars, and bilaterally in the lower splint between the lateral incisors and the canines (Fig. 1). The intraoral elastics were chosen to produce a force magnitude between 200 and 300 g per side according to the stability of the splint and the patient tolerance, while the chin cup was connected to the headgear with a force magnitude ranging between 400 and 600 g per side [10]. The patients were instructed to wear the appliances for 14 h per day and to change the elastics daily [9]. The SEC III protocol was used until a positive overjet of 2 mm was obtained, then the appliances were worn at bedtime for 12 months for maintenance of the obtained correction.

**Fig. 2:**
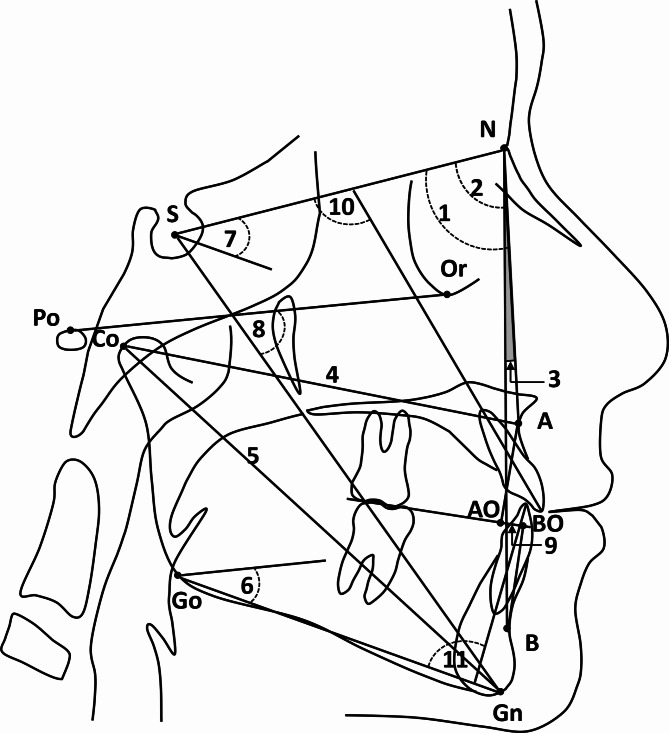
Skeletal and dental cephalometric landmarks and measurements: **(1)** SNA; **(2)** SNB; **(3)** ANB; **(4)** Co-A; **(5)** Co-Gn; **(6)** FMA; **(7)** Go-Gn/SN; **(8)** Y-axis; **(9)** Wits appraisal; **(10)** U1/SN; **(11)** L1/Md. (Refer to Table 1 for definitions of the measured parameters)

**Fig. 3:**
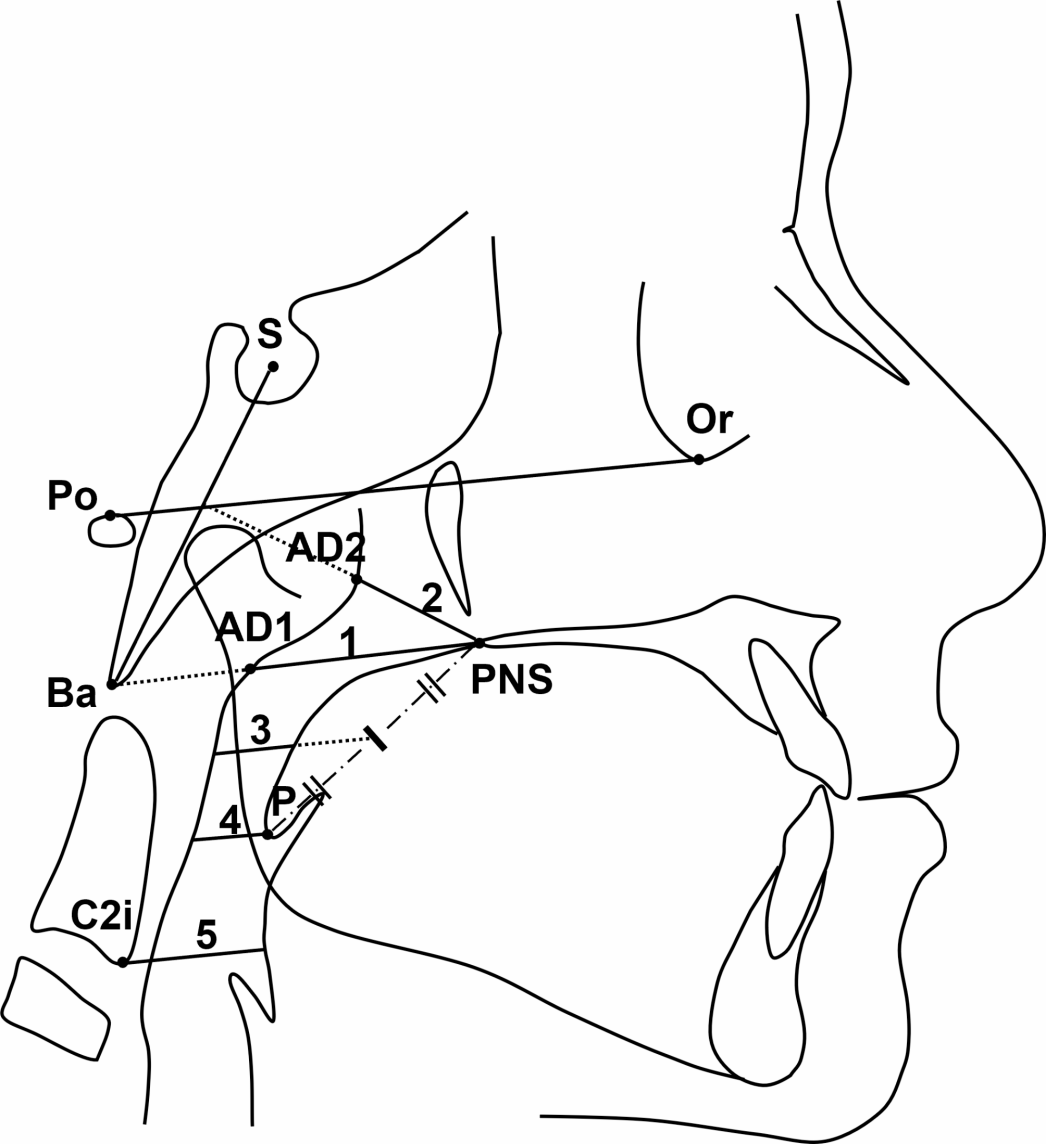
Airway cephalometric landmarks and measurements: **(1)** PNS-AD1; **(2)** PNS-AD2; **(3)** SPS; **(4)** MPS; **(5)** IPS. (Refer to Table 1 for definitions of the measured parameters)

Lateral cephalometric radiographs obtained before and immediately after treatment were manually traced, and cephalometric analysis was performed. During acquisition of the lateral cephalograms, the Frankfurt horizontal plane was oriented parallel to the floor using the cephalostat. All the cephalograms were obtained in maximum intercuspation and natural breathing, and the patients were instructed to avoid swallowing during the acquisition. The cephalometric analysis included skeletal and dental measurements [21], and measurements of the airway dimensions [21, 22] as depicted in Figs. 2 and 3. The measured variables are defined in Table 1.

**Table 1:** Skeletal, dental, and airway cephalometric measurements

Measurement	Definition
SNA	The relationship of the maxilla to the anterior cranial base measured as the angle between the Sella-Nasion (S-N) plane and the Nasion-A point (N-A) line
SNB	The relationship of the mandible to the anterior cranial base measured as the angle between the SN plane and the Nasion-B point (N-B) line
ANB	The relationship of the maxilla to the mandible measured as the angle between the N-A line and the N-B line
Co-A	Maxillary length measured from Condylion (Co) to A point
Co-Gn	Mandibular length measured from Co to Gnathion (Gn)
Mx-Md diff	Maxillo-mandibular differential calculated as the difference between maxillary length (Co-A) and mandibular length (Co-Gn)
FMA	Mandibular plane angle measured between the Frankfurt horizontal plane (line connecting Po and Or) and the mandibular plane (Go-Gn)
Go-Gn/SN	Mandibular plane angle measured between the S-N plane and the mandibular plane (Go-Gn)
Y-axis	The acute angle formed by the intersection of a line from S to Gn with the Frankfurt horizontal plane
Wits appraisal	The distance between two points that are formed by dropping perpendicular lines from A point and B point to the occlusal plane (AO and BO, respectively)
U1/FH	The angle formed by the intersection of the Frankfurt horizontal plane with a line passing through the incisal edge and root apex of the maxillary central incisor
L1/Md	The angle formed by the intersection of the mandibular plane with a line passing through the incisal edge and root apex of the mandibular central incisor
PNS-AD1	distance between PNS and the nearest adenoid tissue measured through the PNS-Ba line
PNS-AD2	distance between PNS and the nearest adenoid tissue measured through a perpendicular line to S-Ba from PNS
SPS	Superior pharyngeal space measured between the posterior pharyngeal wall and the dorsum of the soft palate on a line parallel to the Frankfurt horizontal and running through the middle of the line from posterior nasal spine (PNS) to P (tip of soft palate)
MPS	Middle pharyngeal space measured between the posterior pharyngeal wall and the dorsum of the tongue on a line parallel to Frankfurt horizontal and running through P
IPS	Inferior pharyngeal space measured between the posterior pharyngeal wall and the dorsum of the tongue on a line parallel to the Frankfurt horizontal and running through the most anteroinferior point on the body of the second cervical vertebra (C2i)

### Statistical analysis

Sample size estimation was calculated using G*Power Software version 3.1.9.4 [23] assuming 80% study power and 5% alpha error. Calculations were based on a pilot study conducted on 3 patients [25], using the formula stated below. The pilot data showed that mean ± standard deviation (SD) superior pharyngeal space before and after treatment using SEC III protocol was found to be 21.7 ± 3.1 mm and 19.7 ± 0.6 mm, respectively. Based on comparison of paired means, using two-tailed test with the highest SD to ensure power (effect size = 0.64), the minimum required sample size was 22 patients, which was increased to 24 to make up for procedural problems [24].

1 $$N = {\left( {\frac{{\left( {{t_{1 - \alpha /2}} + {t_{1 - \beta }}} \right)\sigma }}{d}} \right)^2}$$

N is the required sample size, t is the two-tailed T distribution, $\sigma$ represents the standard deviation, $\alpha$ is the given probability value, $\beta$ represents the study power, and d is the effect size [26].

Normality of the data was checked using descriptive statistics, plots (Q-Q plots and histogram), and normality tests. Descriptive statistics were calculated as means and SD for quantitative variables, in addition to frequencies and percentages for qualitative variables. Comparisons of different measurements between T1 and T2 were done using paired samples t-test for normally distributed variables, and Wilcoxon signed rank test for non-normally distributed variables. Mean differences and 95% confidence intervals (CI) were calculated. The significance level was set at p value < 0.05. Data were analysed using Statistical Package for Social Sciences software version 26.0 (IBM Corp., Armonk, NY).

Calibration on cephalometric measurements was done for a single examiner (A.E.). Three weeks after performing the cephalometric measurements, 5 patients were randomly selected using the RANDBETWEEN function in Microsoft 365® Excel® software (Microsoft Corporation, Redmond, WA). Cephalometric analysis of the selected pre-treatment and post-treatment radiographs was repeated by the same researcher and Intraclass Correlation Coefficient (ICC) was calculated.

## Results

Analysis of the data showed that the study sample consisted of 6 males and 18 females, aged 11.21 ± 1.02 years old. Half of the patients were in CS-3 according to CVM, while 4 patients were in CS-2, and 8 were in CS-4. None of the patients were in CS-1. The active treatment using SEC III protocol lasted for 5.75 months on average. The characteristics of the study group are summarized in Table 2.

**Table 2:** Characteristics of the subjects (n = 24)

Variable	
Mean age at T1 (SD), years	11.21 (1.02)
Sex	
Male, n (%)	6 (25%)
Female, n (%)	18 (75%)
Skeletal maturation at T1	
CS1, n (%)	0 (0%)
CS2, n (%)	4 (16.7%)
CS3, n (%)	12 (50%)
CS4, n (%)	8 (33.3%)
Mean duration of treatment (SD), months	5.75 (1.03)

*CS*: Cervical vertebral maturation stage; *SD*: Standard deviation; *T1*: Pre-treatment

The intra-examiner reliability for the cephalometric measurements ranged between 0.762 and 0.998 indicating good to excellent reliability [27]. The data on intra-examiner reliability for the different variables is shown in Table 3.

**Table 3:** Reliability analysis using ICC.

		ICC	95% CI	P value
**Skeletal and dental measurements**	**SNA**	0.956	0.822, 0.989	< 0.001*
	**SNB**	0.762	0.040, 0.941	0.02*
	**FMA**	0.981	0.924, 0.995	< 0.001*
	**GoGn/SN**	0.994	0.975, 0.998	< 0.001*
	**Y-axis**	0.998	0.993, 0.999	< 0.001*
	**Wits appraisal**	0.982	0.929, 0.996	< 0.001*
	**Co-A**	0.994	0.975, 0.998	< 0.001*
	**Co-Gn**	0.946	0.784, 0.987	< 0.001*
	**U1/SN**	0.956	0.828, 0.989	< 0.001*
	**L1/Md**	0.990	0.960, 0.998	< 0.001*
**Airway measurements**	**PNS-AD1**	0.973	0.890, 0.993	< 0.001*
	**PNS-AD2**	0.839	0.784, 0.878	0.02*
	**SPS**	0.963	0.850, 0.991	< 0.001*
	**MPS**	0.951	0.804, 0.988	< 0.001*
	**IPS**	0.954	0.814, 0.989	< 0.001*

ICC: Intraclass Correlation Coefficient, CI: Confidence Interval

*statistically significant at p value < 0.05

The changes in the cephalometric readings from T1 to T2 are shown in Table 4. The nasopharyngeal airway (PNS-AD1 and PNS-AD2) did not change significantly following treatment (p = 0.10 and 0.78, respectively). Similarly, the change in the superior pharyngeal space was not statistically significant (p = 0.39). Contrarily, the middle and inferior pharyngeal space significantly decreased by 1.33 ± 1.97 mm (p = 0.003) and 1.96 ± 2.48 mm (p = 0.001), respectively.

**Table 4:** Pre-treatment and post-treatment cephalometric parameters, and treatment changes (n = 24)

	T1		T2		T2-T1			*p* value
	Mean	SD	Mean	SD	Mean	SD	95% CI	
SNA, °	81.83	2.33	83.04	2.69	1.21	1.48	0.59, 1.83	0.001* ^**a**^
SNB, °	84.04	2.93	82.33	3.17	-1.71	1.92	-2.52, -0.90	< 0.001* ^**a**^
ANB, °	-2.21	2.11	0.71	1.65	2.92	1.50	2.28, 3.56	< 0.001* ^**b**^
Co-A, mm	78.50	8.43	80.79	8.13	2.29	2.69	1.15, 3.43	< 0.001* ^**a**^
Co-Gn, mm	105.00	12.71	106.00	12.30	1.00	2.84	-0.20, 2.20	0.10^a^
Mx-Md diff, mm	26.50	8.28	25.21	9.38	-1.29	2.84	-2.49, -0.09	0.04* ^**a**^
FMA, °	21.54	5.60	22.29	6.15	0.75	1.42	0.15, 1.35	0.02* ^**a**^
GoGn/SN, °	30.55	6.91	31.49	7.32	0.94	1.19	0.46, 1.42	< 0.001* ^**a**^
Y-axis, °	50.15	10.39	51.44	10.63	1.28	1.51	0.68, 1.89	< 0.001* ^**a**^
Wits appraisal, mm	-5.25	3.10	-1.94	1.80	3.31	1.99	2.47, 4.15	< 0.001* ^**b**^
U1/SN, °	113.33	8.33	117.71	6.50	4.38	4.28	2.57, 6.18	< 0.001* ^**a**^
L1/Md, °	95.13	8.12	89.33	9.70	-5.79	6.21	-8.42, -3.17	< 0.001* ^**a**^
PNS-AD1, mm	21.71	3.07	20.63	3.03	-1.08	3.06	-2.38, 0.21	0.10 ^a^
PNS-AD2, mm	18.88	3.88	19.04	4.81	0.17	2.91	-1.06, 1.40	0.78 ^a^
SPS, mm	17.71	3.41	17.13	2.95	-0.58	3.27	-1.96, 0.80	0.39 ^a^
MPS, mm	10.17	2.14	8.83	1.93	-1.33	1.97	-2.17, -0.50	0.003* ^**a**^
IPS, mm	13.75	2.61	11.79	2.32	-1.96	2.48	-3.00, -0.91	0.001* ^**a**^

*CI*: confidence interval; *SD*: standard deviation.; *T1*: pre-treatment; *T2*: post-treatment

*Statistically significant at *p* value < 0.05

^a^ Paired samples t-test was used, ^b^ Wilcoxon signed rank test was used

Treatment using the SEC III protocol resulted in a significant increase in SNA angle (Mean change = 1.21 ± 1.48 degrees; p = 0.001), a significant decrease in SNB angle (Mean change= -1.71 ± 1.92 degrees; p < 0.001), with a resultant significant increase in ANB angle (Mean change = 2.92 ± 1.50 degrees; p < 0.001). In addition, the increase in the mandibular plane angle (FMA: 0.75 ± 1.42 degrees and GoGn/SN: 0.94 ± 1.19 degrees) was statistically significant (p = 0.02 and p < 0.001, respectively). The maxillary length significantly increased from 78.50 ± 8.43 mm to 80.79 ± 8.13 mm (p < 0.001). On the other hand, the mandibular length did not change significantly (p = 0.10). Y-axis increased significantly by 1.28 ± 1.51 degrees.

The increase in Wits appraisal following SEC III protocol was statistically significant (Mean change = 3.31 ± 1.99 mm; p < 0.001). The maxillary incisors significantly proclined from T1 to T2 (Mean change = 4.38 ± 4.28 degrees; p < 0.001), while the mandibular incisors significantly retroclined (Mean change = -5.79 ± 6.21 degrees; p < 0.001).

## Discussion

Skeletal class III malocclusion is one of the most challenging situations faced by orthodontists. Early interception of the problem may evade more invasive surgical treatment options later in life [6]. The SEC III protocol that was investigated in the current study is one of the treatment approaches employed for correction of class III discrepancies [9, 10, 16, 20]. The SEC III protocol constitutes the use of splints, class III elastics, and chin cup to treat skeletal class III malocclusion. The use of flat occlusal splints aids in the correction of the class III problem by eliminating the intercuspation, hence facilitating the orthopaedic effects of the class III elastics [9], while the addition of a chin cup aims to minimize the clockwise rotation of the mandible [20]. Patients with craniofacial malformations were excluded because previous research has shown that the airway dimensions of cleft lip/palate patients are different from non-cleft patients [28]. Patients in the pre-pubertal or circumpubertal CVM stages were included in the current study because previous research has shown that favourable dentoskeletal results were obtained using a modified SEC III protocol, with no difference between the two stages [29].

In the current study, a significant improvement in the maxillary length (Co-A), SNA angle, ANB angle, and Wits appraisal was observed. Furthermore, the change in the mandibular length (Co-Gn) was not statistically significant suggesting good control of the mandibular growth as a result of treatment. Analogous treatment effects have been reported previously [9, 10]. The significant decrease in the SNB angle in the current study may be related to clockwise rotation of the mandible, which is evident from the statistically significant, though not clinically significant, increase in the mandibular plane angle. Additionally, the increase in the Y-axis angle is suggestive of a downwards and backwards positioning of the chin following treatment using SEC III protocol.

The skeletal changes provided by the SEC III protocol may affect the airway dimensions since the size of the jaws has been shown to correlate with the size of the pharyngeal airway [12]. A recent systematic review aimed to investigate the changes in airway dimensions following treatment of skeletal class III malocclusion using different orthopaedic appliances [30]. However, none of the studies included in the review considered the effect of the SEC III protocol on the airway dimensions [30]. Hence, the primary aim of the current study was to evaluate the airway changes resulting from the use of the SEC III protocol to treat skeletal class III malocclusion in growing patients.

In the current study, the middle and inferior pharyngeal space (retroglossal airway) significantly decreased following treatment using the SEC III protocol. In contrast, previous research investigating treatment of skeletal class III using a facemask combined with a removable lower bite block [31] or combined with a bonded maxillary expander [32, 33] or banded maxillary expander [21] did not find any significant changes in the oropharyngeal dimensions. The decrease in the retroglossal dimensions in the current study may be related to the statistically significant backward rotation of the mandible that took place. A previous study has shown that posterior displacement of the mandible results in a reduction of the airway dimensions, which was attributed to the backward positioning of the tongue and hyoid bone that accompany the mandibular rotation [34]. A decrease in the retroglossal airway dimensions may possibly affect breathing, hence further investigations regarding the changes in the breathing mode and the resistance to airflow following treatment are warranted to better clarify the clinical significance of the obtained results.

The nasopharyngeal airway, delineated by PNS-AD1 and PNS-AD2, and the superior pharyngeal space (retropalatal airway) did not change significantly following treatment in the present study. Analogous results were formerly obtained using a chin cup with a removable lower bite plane to treat skeletal class III in prepubertal patients [32]. The lack of significant changes in the nasopharyngeal and retropalatal airways as opposed to the retroglossal airway may be related to the skeletal effect of the chin cup, which mainly affects the mandible [32]. Likewise, previous research investigating the effect of facemask on the pharyngeal dimensions found no significant changes in the upper airway dimensions despite improvement in the skeletal dimensions of the maxilla [31]. This may be attributed to the gradual increase in the size of the adenoids that normally takes place at the onset of puberty [35]. All the patients in the current study were in the pre-pubertal or circum-pubertal stages at the start of treatment, hence an increase in the size of the adenoids was expected during the treatment period, which may have offset any changes that took place in the size of the nasopharynx as a result of treatment.

Contrary to the current study, treatment of skeletal class III using facemask combined with rapid maxillary expansion [21, 32] or alternate rapid maxillary expansion and constriction protocol [36] was previously shown to result in a significant increase in the nasopharynx or retropalatal dimensions. A possible reason for the conflicting results is that a smaller increase in the skeletal maxillary dimensions was obtained in the current study compared to the aforementioned studies [21, 32, 36]. In addition, the use of a maxillary expander may have favourably affected the airway dimensions [37], especially considering that maxillary expansion significantly increases the skeletal transverse dimension [38].

Correction of the class III discrepancy in the current study was obtained within 5.75 ± 1.03 months, which is less than the previously reported durations. Perillo et al. [10] stated that the correction of the skeletal discrepancy using SEC III protocol required 1 year on average, while Ferro et al. [9] reported that the active treatment lasted on average for 4 years, with a range of 0.3 to 10 years. The shorter duration in the current study may possibly be related to better patient compliance. However, verification of compliance was not possible due to the retrospective study design. Another factor that might have affected the duration of active treatment is the wide age range (4 to 15 years) that was reported by Ferro et al. [9].

In addition, attainment of a positive overjet within a short period of time may have been facilitated by the dental compensation that took place with treatment. Notable proclination of the maxillary incisors and retroclination of the mandibular incisors was reported in the current study. Such dentoalveolar compensation is likely related to the use of class III elastics with the occlusal splints [39]. However, although the change in the inclination of the incisors contributed to the rapid correction of the class III malocclusion, it might affect the stability of the obtained correction in the long run [9]. Hence, long-term studies are recommended to evaluate the stability of the treatment outcomes.

## Limitations

A limitation of the current study is that no control group was included. The assessment of an untreated group would have allowed evaluation of changes not pertaining to the SEC III protocol; however, this was not feasible due to ethical concerns. Additionally, two-dimensional lateral cephalometric radiographs were used to evaluate the treatment changes in a three-dimensional structure, which may have overlooked substantial findings [40]. Nevertheless, lateral cephalograms are routinely obtained for orthodontic patients, hence exposure of young patients to additional radiation was avoided. Another shortcoming is that the correction of the class III problem was discernible in the post-treatment radiographs, thus blinding of the outcome assessor was not possible. Moreover, due to the retrospective nature of the study, it was difficult to report on the patients’ compliance with the appliances, the side effects resulting from the use of the appliances, or the change in airflow following treatment.

Future studies with a prospective design, a larger sample size and long-term follow-up should be conducted. The research should focus on evaluating the three-dimensional changes of the airway, assessing the patient-reported experiences, and performing functional analysis of the airway following SEC III protocol. It is also recommended to conduct more studies to compare the effects of the SEC III protocol with the effects of other appliances used to treat skeletal class III malocclusion [41, 42], in non-cleft and in cleft lip/palate patients [28].

## Conclusions

Within the limitations of the study, it can be concluded that:

- Early class III correction using SEC III protocol did not affect the nasopharyngeal and retropalatal airway dimension.
- Early class III correction using SEC III protocol reduced the retroglossal airway dimensions.
- Correction of the class III dentoskeletal relationship using SEC III protocol was obtained through both skeletal and dental changes.

## Data Availability

The datasets used and/or analysed during the current study are available from the corresponding author upon reasonable request.

## Abbreviations

CS: Cervical stage
CVM: Cervical Vertebral Maturation
ICC: Intraclass Correlation Coefficient
SD: Standard Deviation
SEC III: Splints, Class III Elastics, and Chin cup
